# 6DoF Object Pose and Focal Length Estimation from Single RGB Images in Uncontrolled Environments

**DOI:** 10.3390/s24175474

**Published:** 2024-08-23

**Authors:** Mayura Manawadu, Soon-Yong Park

**Affiliations:** Graduate School of Electronic and Electrical Engineering, Kyungpook National University, Daegu 41566, Republic of Korea; mayuramanawadu@knu.ac.kr

**Keywords:** 6DoF, pose estimation, focal length, uncontrolled RGB images, XR

## Abstract

Accurate 6DoF (degrees of freedom) pose and focal length estimation are important in extended reality (XR) applications, enabling precise object alignment and projection scaling, thereby enhancing user experiences. This study focuses on improving 6DoF pose estimation using single RGB images of unknown camera metadata. Estimating the 6DoF pose and focal length from an uncontrolled RGB image, obtained from the internet, is challenging because it often lacks crucial metadata. Existing methods such as FocalPose and Focalpose++ have made progress in this domain but still face challenges due to the projection scale ambiguity between the translation of an object along the z-axis ($t_{z}$) and the camera’s focal length. To overcome this, we propose a two-stage strategy that decouples the projection scaling ambiguity in the estimation of z-axis translation and focal length. In the first stage, $t_{z}$ is set arbitrarily, and we predict all the other pose parameters and focal length relative to the fixed $t_{z}$. In the second stage, we predict the true value of $t_{z}$ while scaling the focal length based on the $t_{z}$ update. The proposed two-stage method reduces projection scale ambiguity in RGB images and improves pose estimation accuracy. The iterative update rules constrained to the first stage and tailored loss functions including Huber loss in the second stage enhance the accuracy in both 6DoF pose and focal length estimation. Experimental results using benchmark datasets show significant improvements in terms of median rotation and translation errors, as well as better projection accuracy compared to the existing state-of-the-art methods. In an evaluation across the Pix3D datasets (chair, sofa, table, and bed), the proposed two-stage method improves projection accuracy by approximately 7.19%. Additionally, the incorporation of Huber loss resulted in a significant reduction in translation and focal length errors by 20.27% and 6.65%, respectively, in comparison to the Focalpose++ method.

## 1. Introduction

Precise real and virtual object registration is of importance in extended reality (XR) applications for creating immersive and interactive user experiences. It allows seamless integration of virtual objects with real environments. To ensure precise alignment of virtual objects in the real environment, the accurate 6DoF pose information of the objects and camera parameters are required. The 6DoF pose estimation involves determining the position and orientation of an object in 3D space. Consequently, it has become an increasingly important research topic among the computer vision community.

Most existing methods for 6DoF pose estimation require calibrated intrinsic camera metadata to achieve high precision [1,2,3,4,5,6,7,8,9]. However, this task becomes particularly challenging when dealing with uncontrolled ’in the wild’ RGB images, which often lack metadata such as camera focal length. These include images obtained from the internet, educational books, newspapers, or photos taken in environments where camera settings are unknown. While many consumer-grade cameras and smartphones include metadata in their EXIF data, there are many scenarios where this information may not be available due to privacy settings or because image editing software strips away this metadata. Additionally, images captured as screenshots often lose their original data. The absence of camera metadata can affect precise object overlay, significantly impacting both the accuracy and reliability of 6DoF pose estimation. When camera metadata are unavailable, the estimation process must compensate for the lack of focal length, which is crucial for accurate scaling and depth perception. This absence can lead to errors in determining the correct size and position of virtual objects relative to the real-world scene, resulting in a less immersive and convincing user experience.

Solutions that can precisely register virtual objects to uncontrolled real images are essential in many applications where accurate 6DoF poses of these objects are required, but challenging due to the lack of camera information. For example, in AR applications for enhancing tourist experiences at historical sites, images from the internet are often used to create virtual reconstructions. Without reliable camera information, conventional methods struggle to overlay and scale the virtual objects to the images, reducing the immersive experience. In real estate and interior design, accurate pose estimation ensures that virtual furniture fits correctly in real spaces. Photos taken by potential buyers during property visits are often compressed for easier sharing, which removes important metadata such as focal length. This loss affects the precise placement of virtual objects, which is important for decision-making and visualization. In educational settings, AR transforms learning experiences by projecting interactive content onto textbook pages. Printed images in textbooks do not provide readable metadata such as focal length. To overlay these virtual objects, accurate 6DoF pose estimation can enable the interactive 3D contents to be projected on physical pages. Additionally, in application areas such as 3D multi-object tracking and detection, methods of estimating camera matrices or adaptive updates are beneficial. For instance, methods in [10,11] demonstrate how accurate camera matrix estimation is critical for improving tracking performance in complex environments. Knowing camera intrinsic parameters can improve the initialization and re-identification of tracks, handle occlusions better, and adapt to changes in camera configurations. These applications highlight the importance of developing reliable solutions for 6DoF pose estimation and focal length estimation to enhance the immersive experience.

Various methods have been introduced for 6-DoF pose estimation, primarily using RGB-D [1,2,3] and RGB images. RGB-D methods leverage the depth information available, making the estimation process less reliant on accurate focal length, as the depth information can be utilized in pose determination. DenseFusion [3] integrates RGB and depth data for 6-DoF pose estimation, demonstrating high robustness in cluttered and occluded scenes. On the other hand, RGB-based methods [4,5,6,7,8,9] mostly rely on focal length information, which can result in significant challenges when intrinsic focal length is unavailable, as it influences the scaling and depth of the objects in the image.

Several investigations [9,12,13] have been introduced to jointly estimate the 6D pose and the camera’s focal length from a single RGB image. Among those works, FocalPose [13] by Ponimatkin et al. and Focalpose++ [14] by Cifka et al. can be considered as the existing state-of-the-art work related to this domain. It employs a pose and focal length update rule using a render-and-compare approach, showing improved results on benchmark datasets. Extending the FocalPose [13], FocalPose++ [14] improves the translation update rules instead of the approximation used in FocalPose. However, simultaneous estimation of the focal length of a camera and z-axis translation of an object in the update rules of both [13,14] affects the scaling of the projected object rendering on the image. This challenge arises from the correlation inherent to the scaling property between internal focal length and external z-axis translation within the perspective projection of the pinhole camera model. Hence, the simultaneous estimation of these two correlated parameters causing the projection scale ambiguity should be addressed.

The proposed work addresses this issue by presenting a two-stage strategy that mitigates the projection scale ambiguity by decomposing the simultaneous estimation of focal length and z-axis translation ($t_{z}$). The contributions of this work are as follows: We introduce a two-stage strategy for 6DoF pose and focal length estimation from single RGB images taken in uncontrolled environments without camera metadata. The proposed approach addresses projection scale ambiguity by separating the estimation of the camera’s focal length and the object’s z-axis translation. In the initial stage, ($t_{z}$) translation is fixed to an arbitrary constant value, simplifying the estimation process for the other pose parameters ($t_{x},t_{y},r_{1},r_{2},r_{3}$, and focal length (f)). Experimental results show that this initial simplification enhances the precision of estimations and provides a reliable foundation for subsequent refinements in Stage II. In the second stage, the value of the previously set ($t_{z}$) translation is predicted, and the focal length is scaled relative to the predicted ($t_{z}$) value. Additionally, from this work, the Huber loss of $t_{z}$ is introduced to the loss function of the second stage to further enhance the estimation of the initially fixed $t_{z}$, resulting in a 2.79% decrease in translation error and a 0.41% decrease in focal length error on average. The effects of different loss functions are discussed in the ablation studies, highlighting their impact on performance. This work also shows the effect of selecting different arbitrary values for $t_{z}$ in the first stage and how this choice impacts pose estimation accuracy. Additionally, it examines the divergence issue when applying a refiner in the second stage. This two-stage approach resolves previous uncertainties and demonstrates an improvement of projection accuracy by 7.19% over the existing methods, as presented by the experimental results. These contributions are useful across various fields, including XR, robotics, and 3D object tracking, enhancing the integration of virtual objects with real-world environments and improving user experiences.

## 2. Related Works

Pose estimation is fundamental in numerous computer vision applications, especially for augmented reality (AR) and robot vision. Among the numerous efforts made by the computer vision research community [15,16], this section categorizes and presents the approaches based on classical and deep learning-based methods. This classification is chosen because it highlights the evolution from conventional techniques to modern deep learning methods, illustrating the advancements in accuracy and robustness. While this classification may not cover all types of related research, such as event-based camera systems [17,18], it provides a high-level overview of the developments in 6D pose estimation research.

### 2.1. Classical Approaches

Classical pose estimation methods, developed before deep learning, are still prominent and effective in computer vision. Techniques such as template matching, descriptor-based, and feature-based algorithms have unique strengths and continue to achieve significant results.

#### 2.1.1. Template Matching

Template matching algorithms use reference images with distinctive features [19] to create standard templates for objects. These templates are then compared to the target image to find the best match and estimate the object’s pose. Methods, such as the iterative closest point (ICP) [20], can be used to improve the alignment accuracy.

Template matching is effective for objects with minimal texture, relying on global features. However, these algorithms are highly sensitive to variations in illumination and object posture, which can significantly impact their performance. Changes in lighting can alter the appearance of the object, causing mismatches between the template and the target image due to differences in brightness, shadows, and reflections. Variations in object posture, such as rotation or tilting, can lead to incorrect pose estimation if the object appears differently from how it is represented in the template. Hence, it requires many manually created templates, which is time-consuming [21,22,23]. Recent advancements have improved computational efficiency and robustness. For example, Vock et al. [24] developed a fast method for processing 3D point clouds using a new edge detection and sampling strategy, significantly increasing speed. However, this method could benefit from improvements to handle uneven point densities.

Similarly, Reinbacher et al. [25] proposed a method based on hierarchical silhouette matching and unsupervised clustering, reducing the matching time by 80% compared to exhaustive matching and demonstrating scalability. This method is robust for smooth, untextured, and slightly transparent objects. However, the accuracy with symmetrical objects could be improved by using multiple views to enhance rotational accuracy.

#### 2.1.2. Descriptor-Based Techniques

Descriptor-based methods are essential for 6DoF pose estimation, encoding an object’s local geometry. Point feature histograms (PFHs) [26,27] and fast point feature histograms (FPFH) [28] create multi-dimensional histograms based on spatial differences, with FPFHs being more efficient. SHOT (signature of histograms of orientations) [29] and Spin Images [30] describe local surfaces effectively.

Recent advancements combine descriptor-based methods with deep learning to improve feature extraction and pose estimation. For example, the BOLD3D descriptor [31] uses edge information for robust pose detection, enhancing accuracy in challenging environments. Future research could further integrate BOLD3D with other descriptors for better performance.

#### 2.1.3. Feature-Based Methods

Feature-based object detection in 6D pose estimation is based on identifying features like edges, interest points, and corners. These features are used by detection algorithms to estimate an object’s pose by comparing them to reference features, accurately determining the position and orientation of objects in a scene.

Yoon et al. [32] presented a fast-tracking algorithm for real-time pose estimation of industrial objects using geometric features in a stereo vision setup, achieving high accuracy and robustness at 60 fps. Further enhancements could include adaptive lighting techniques and alternative feature shapes for complex industrial scenarios.

Seppälä et al. [33] introduced a tool for feature-based object detection and pose estimation using 3D point clouds and CAD models. This method improves accuracy and efficiency in manufacturing environments by matching measurable features from CAD models to 3D point clouds. Future developments could integrate advanced 3D sensor data processing and more flexible software.

Teney et al. [34] proposed a unified method for detection, localization, and continuous pose estimation using probabilistic models and kernel density estimation, which is robust with non-textured objects. Improvements in computational efficiency and data augmentation strategies could enhance scalability for real-time applications.

### 2.2. Deep Learning Based Approaches

Deep learning has significantly improved 6D object pose estimation using convolutional neural networks (CNNs) to learn robust feature representations from RGB and RGB-D images. These methods, despite being data-dependent, enhance accuracy and robustness by an end-to-end training of the CNNs. They can be broadly categorized into RGB-D image-based methods and RGB image-based methods.

#### 2.2.1. RGB-D Image-Based Approaches

Combining RGB images and depth information from RGB-D sensors [35,36] enhances 6DoF object pose estimation by providing rich geometric details along with color and texture, facilitating to overcome occlusions, varying lighting conditions, and cluttered backgrounds.

DenseFusion [3], developed by Wang et al., integrates RGB and depth data for 6D object pose estimation. It processes RGB and depth data separately and then fuses them at a pixel level, achieving high robustness and accuracy in cluttered and occluded scenes. Enhancements could focus on optimizing the fusion process and better handling varying depth data quality.

Balntas et al. [1] introduced a method for pose-guided RGB-D feature learning, using object poses to guide robust feature learning and improve pose recognition accuracy. Potential improvements include symmetry-aware learning and optimized computational efficiency.

Tian et al. [2] proposed a method for robust 6D object pose estimation using densely extracted RGB-D features, demonstrating robustness to occlusions and clutter. Further research could optimize regression strategies and enhance real-time capabilities.

#### 2.2.2. RGB Image-Based Approaches

One of the significant works in this area is DeepIM [4], developed by Li et al. DeepIM iteratively refines initial pose estimates by aligning a rendered image of the object with the observed image. Using a CNN, it predicts small adjustments to the pose in each iteration, making it robust to initial pose errors and suitable for various objects.

Building on DeepIM, CosyPose [5], by Labbe et al., extends the iterative approach to multiple views and objects, estimating the 6D poses of several objects in a scene from multiple RGB images. It performs well on benchmarks but can be computationally intensive. Efforts to speed up these steps and improve scalability would be beneficial.

Park et al. [9] proposed a pipeline for 4-DoF pose estimation using RGB images and CAD models from the ShapeNet dataset. Their method detects the object, estimates a 3-DoF rotational pose with the PoseContrast network [37], and determines the 1-DoF focal length by comparing 2D renderings of CAD candidates. This approach simplifies pose estimation, making it efficient and suitable for extended reality (XR) applications. Enhancing robustness to handle a broader range of objects would be beneficial.

Most previous works assume that camera intrinsic parameters are known, limiting the problem to estimating only the 6DoF pose parameters. However, when dealing with ’in the wild’ RGB images, the complexity increases as it is required to estimate the camera’s intrinsic focal length as well. To address this, Ponimatkin et al. introduced FocalPose [13], which estimates both the 6D pose and the camera focal length from a single RGB image. Using a render-and-compare strategy, [13] handles uncalibrated RGB images with unknown focal lengths. While earlier works such as GCVNet [12] and GP2C [38] have also focused on joint estimation, Focalpose [13] and Focalpose++ [14] can be considered as the state-of-the-art. GP2C is limited in that it cannot be trained end-to-end as it relies on a separate non-differentiable optimizer, and GCVNet’s results are limited by the approximation of the PnPf solver used for differentiability. Built on the CosyPose approach, Ref. [13] uses a neural renderer for estimating 6DoF pose and focal length. Although it introduces a loss function that separates the effects of focal length and pose, producing promising results, room for improvement was identified in the pose update rules of existing methods. Specifically, the coupling of focal length and z-axis translation ($t_{z}$) affects object scaling, suggesting potential areas for further refinement. A most recent work, FocalPose++ [14], is an extension of FocalPose, improving the update rules of $t_{x}$ and $t_{y}$ by incorporating the focal lengths of two consecutive iterations according to the principles of perspective projection. Additionally, it also explores using parametric distribution for synthetic datasets with real datasets [14]. Despite these improvements, the coupling between $t_{z}$ and focal length persists.

The proposed approach from this study addresses the scaling issue by decoupling these parameters and using a two-stage process to simplify estimation and improve accuracy. Initially, the z-axis translation is fixed ($t_{z}$) to a constant value, allowing for more reliable prediction of other pose parameters, including focal length (*f*). In the second stage, fixed z-axis translation from Stage I is predicted while scaling the focal length relative to the update of $t_{z}$. Experimental results show that this strategy overcomes this projection scale ambiguity identified in [13], providing robust and accurate 6D pose estimation from single RGB images. Building on our work [39], this study introduces a Huber loss of translation of z-axis to the loss function to further enhance the results. While the improvements in some metrics are comparable, our results still demonstrate improvements in several metrics as given in the Section 4, and consistently outperform the methods described in [13,14].

## 3. Methodology

### 3.1. Motivation

This section explains the existing projection scale ambiguity in the simultaneous prediction of 6DoF pose and focal length. It also describes the strategic approach of the proposed work in addressing ambiguity by fixing and re-scaling the correlated parameters.

#### 3.1.1. Projection Scale Ambiguity in Perspective Projection of Pinhole Camera Model

**Figure 1:**
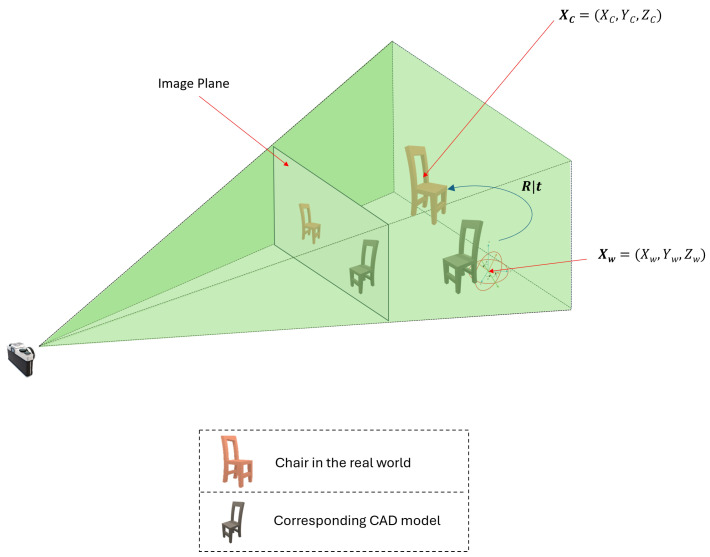
Projection of an object onto the image plane of a pinhole camera using perspective projection.

For the experiments, the perspective camera projection for the pinhole camera model is devised as illustrated in Figure 1. The objective is to determine the 6DoF (six degrees of freedom) pose of the object that appears in an RGB image that does not have focal length details.

Consider the scenario given in Figure 1. The brown-colored chair represents the real-world object positioned in the world coordinate system that produced the image. Initially, a CAD model (black color) is placed at $X_{w}$, the origin (coordinates represented by $X_{w}$, $Y_{w}$, $Z_{w}$), as described by Equation (1), as follows:

(1) $$X_{w}=\left[\begin{array}{c} X_{w}\\ Y_{w}\\ Z_{w} \end{array}\right]$$

As mentioned above, given only an RGB image (where the real-world chair is the object that formed the image) the goal of the proposed work is to predict the position and orientation (6DoF) of the real-world chair from the image. To achieve this, a CAD model is used as a reference, adjusting its position and orientation using a render-and-compare strategy. This process iteratively continues until the CAD model is accurately aligned with the real-world chair. Precise alignment of the CAD model’s rendering on the image of the chair indicates correct positioning at the target 6DoF and focal length.

To achieve this, rotation R and translation t are applied to the CAD model so that it aligns with the real-world chair. This process involves applying an extrinsic transformation to the CAD model, transforming its reference frame to the camera coordinate system. The rotation matrix R and translation vector t are defined by Equations (2) and (3), respectively. The terms $r_{11}$, $r_{12}$, and $r_{13}$ represent the rotational components along the *x*, *y* and *z* axes. Similarly, $t_{x}$, $t_{y}$, and $t_{z}$ represent the translational components along the *x*, *y*, and *z* axes.

(2) $$R=\left[\begin{array}{c} r_{1}^{T}\\ r_{2}^{T}\\ r_{3}^{T} \end{array}\right]=\left[\begin{array}{ccc} r_{11} & r_{12} & r_{13}\\ r_{21} & r_{22} & r_{23}\\ r_{31} & r_{32} & r_{33} \end{array}\right]$$

(3) $$t={\left[\begin{array}{ccc} t_{x} & t_{y} & t_{z} \end{array}\right]}^{T}$$

Hence, this transformation will convert the coordinates of the CAD model to the camera coordinate system $X_{c}$, as given by Equation (4). The terms $X_{c}$, $Y_{c}$, and $Z_{c}$ represent the camera coordinates.

(4) $$X_{c}=\left[\begin{array}{c} X_{c}\\ Y_{c}\\ Z_{c} \end{array}\right]=\left[\begin{array}{c} r_{1}^{T}X_{w}+t_{x}\\ r_{2}^{T}X_{w}+t_{y}\\ r_{3}^{T}X_{w}+t_{z} \end{array}\right]$$

The coordinates in the camera coordinate system must be multiplied by the intrinsic matrix to obtain the coordinates in the image plane. This projection results in a new point in image space represented by u,v,w in homogeneous coordinates. These coordinates must be converted into Cartesian Coordinates to be usable, as given by Equations (5)–(7). Term *f* in these equations represents the focal length.

(5) $$\left[\begin{array}{c} u\\ v\\ w \end{array}\right]=\left[\begin{array}{ccc} f & 0 & 0\\ 0 & f & 0\\ 0 & 0 & 1 \end{array}\right]\left[\begin{array}{c} X_{c}\\ Y_{c}\\ Z_{c} \end{array}\right]$$

(6) $$x=u/w=\frac{f}{Z_{c}}X_{c}$$

(7) $$y=v/w=\frac{f}{Z_{c}}Y_{c}$$

Substituting the value of $Z_{c}$ in Equation (4) to Equations (6) and (7) results in the following:

(8) $$x=\frac{f}{r_{3}^{T}X_{w}+t_{z}}X_{c}$$

(9) $$y=\frac{f}{r_{3}^{T}X_{w}+t_{z}}Y_{c}$$

Given the *x* and *y* values of the image space coordinates, which are obtained from the single RGB image, the goal of the proposed work is to estimate the camera’s focal length and six degrees of freedom (6DoF) from that single RGB image. The FocalPose approach by Ponimatkin et al. [13] seeks to address this challenge by simultaneously predicting all these parameters using the update rules. However, this simultaneous estimation introduces a noticeable scaling ambiguity due to the interdependence of the focal length and the z-axis translation ($t_{z}$). This ambiguity critically affects the projection scale of objects onto the image plane, leading to issues in rendering accuracy and yielding ambiguous results in pose estimation. Accurate rendering is important for practical applications such as 3D modeling and augmented reality, where precise depth representation is essential.

#### 3.1.2. Decoupling Ambiguity in Projection Scale by Fixing One Correlated Parameter to an Arbitrary Constant

Given the complexity introduced by the simultaneous estimation of the focal length and z-axis translation, the proposed method simplifies the problem by fixing $t_{z}$ to an arbitrary constant. This decision is driven by practical and theoretical considerations: fixing $t_{z}$ reduces the degrees of freedom in the estimation problem and provides a controlled base from which to accurately re-calibrate other parameters. While it is possible to fix the focal length instead, choosing $t_{z}$ is often more intuitive and aligns with practical imaging scenarios where depth can vary widely but is generally within a predictable range. This approach enables leveraging approximate depth information that might be available or inferred from the context, making the setup more adaptable to real-world applications.

**Figure 2:**
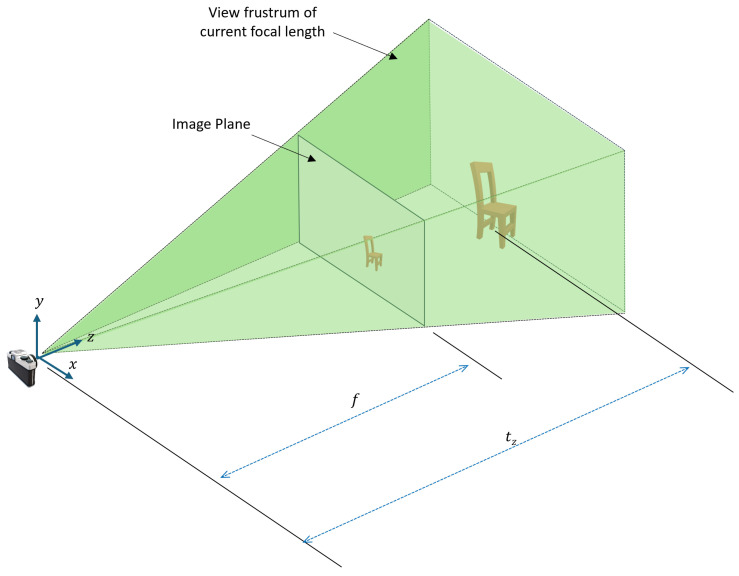
Initial position and orientation of the real-world chair and the image plane based on ground truth values.

This section discusses the phenomena of fixing a correlated parameter and how to tackle the problem strategically. Figure 2 shows the position and orientation of a real-world chair resulting in the formation of a given RGB image.

Setting $t_{z}$ to an arbitrary constant $z_{arb}$ impacts the projection scale of the image. Fixing $t_{z}$ changes the camera coordinates to $X_{c}^{\prime }$ as shown in Equation (10), as follows:

(10) $$X_{c}^{\prime }=\left[\begin{array}{c} X_{c}\\ Y_{c}\\ Z_{c}^{\prime } \end{array}\right]=\left[\begin{array}{c} r_{1}^{T}X_{w}+t_{x}\\ r_{2}^{T}X_{w}+t_{y}\\ r_{3}^{T}X_{w}+z_{arb} \end{array}\right]$$

Thus, this change in the camera coordinate system results in a change in the image space coordinates *x* and *y* to $x^{\prime }$ and $y^{\prime }$ as given by Equations (11) and (12):

(11) $$x^{\prime }=\frac{f}{Z_{c}^{\prime }}X_{c}=\frac{f}{r_{3}^{T}X_{w}+z_{arb}}X_{c}$$

(12) $$y^{\prime }=\frac{f}{Z_{c}^{\prime }}Y_{c}=\frac{f}{r_{3}^{T}X_{w}+z_{arb}}X_{c}$$

**Figure 3:**
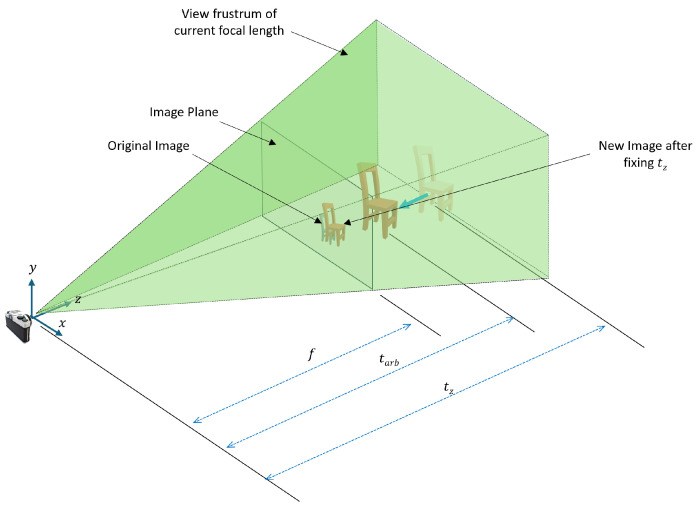
Change of the projection scale of the image after setting $t_{z}$ to an arbitrary value.

By setting $t_{z}$ to a constant value, the perceived distance between the camera and the object along the z-axis is changed, which inherently changes the scale of the object in the 2D image plane. This effect is demonstrated in Figure 3.

The z-axis component directly influences the scaling factor in the perspective projection formula. Objects closer to the camera appear larger, and those further away appear smaller. From Equations (6) and (7), the scale change can be represented by Equation (13), where $S_{f}$ represents the scaling factor. In this equation, only the *X* axis coordinate is considered for simplicity in the explanation:

(13) $$S_{f}=\frac{x^{\prime }}{x}=\frac{r_{3}^{T}X_{w}+t_{z}}{r_{3}^{T}X_{w}+z_{arb}}$$

To prevent this scaling issue and ensure $x^{\prime }\to x$, the focal length must be adjusted accordingly. This adjustment is necessary because the focal length is correlated with $t_{z}$ in terms of image scaling. To make $x^{\prime }\to x$, the focal length is adjusted according to Equation (14):

(14) $$x^{\prime }\frac{x}{x^{\prime }}=x^{\prime }\frac{1}{S_{f}}=\frac{f\frac{1}{S_{f}}}{r_{3}^{T}X_{w}+t_{z}}X_{c}=\frac{f_{new}}{r_{3}^{T}X_{w}+t_{z}}X_{c}$$

The CAD model is represented by a point cloud; hence, it results in numerous $X_{w}$ coordinates for each point in the point cloud. Calculating the focal length change $f_{\mathrm{new}}$ for each point using the scale ratio from Equation (13) is computationally expensive. Therefore, the weak perspective projection model [40], which sets $r_{3}^{T}X_{w}=0$, is used. The weak perspective projection model simplifies the process by assuming that depth differences are small compared to the distance from the camera, effectively flattening the scene so all points are at the same distance. By setting $r_{3}^{T}X_{w}=0$, division individually per each coordinate is avoided, which applies the same scale factor across the entire image, simplifying the math. Using weak perspective projection maintains a consistent scale throughout the image plane.

In consequence, using the weak perspective projection, Equation (13) can be simplified to Equation (15):

(15) $$S_{f}=\frac{t_{z}}{z_{arb}}$$

Thus, to compensate for the altered projection scale caused by fixing $t_{z}$, it is necessary to adjust the initial focal length *f* to $f_{new}$ using the scaling factor as shown in Equation (16)

(16) $$f_{\mathrm{new}}=\frac{1}{S_{f}}\times f=\left(\frac{z_{arb}}{t_{z}}\right)\times f$$

**Figure 4:**
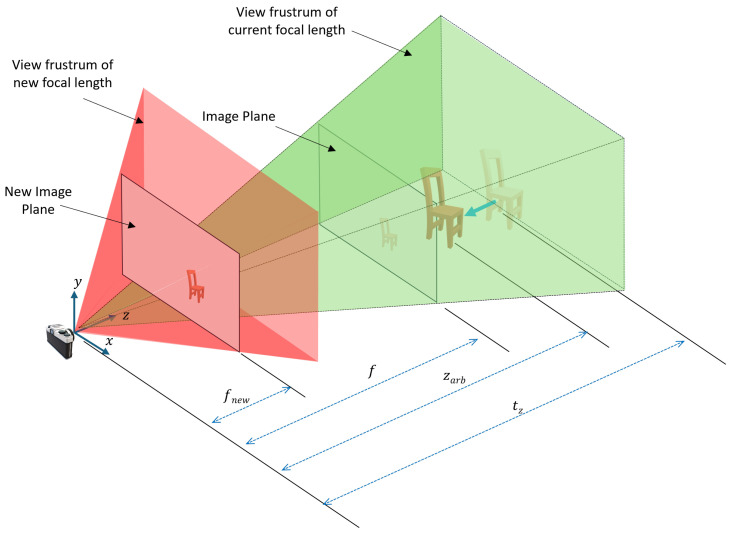
Obtaining the same projection size of the chair by re-scaling the focal length relative to the adjustment of $t_{z}$.

This overall adjustment results in projecting the image with the same scale as in the original image, however, in a new image plane with updated focal length as given by Figure 4. This adjustment of the focal length is crucial for ensuring that the projection on the image plane reflects the true scale of the scene as observed in the original RGB image. Without this adjustment, objects could appear incorrectly scaled or positioned, leading to significant errors in applications requiring high precision, such as augmented reality and 3D modeling.

While the initial stages involve fixing $t_{z}$ to simplify the problem, the ultimate goal is to predict the original $t_{z}$ value accurately. This prediction is achieved using a two-stage approach.

### 3.2. Two-Stage Approach for 6DoF Pose and Focal Length Prediction

**Figure 5:**
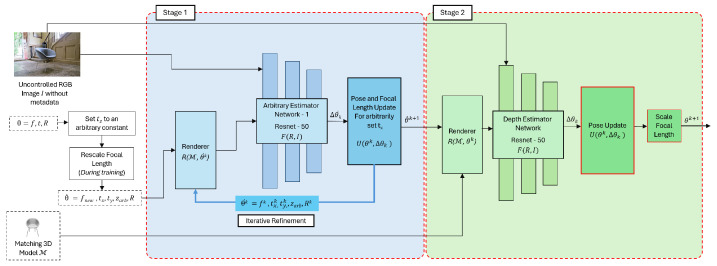
Two-stage approach for predicting the 6DoF pose estimation and focal length from a single uncontrolled RGB image.

Following the strategy to simplify the estimation problem by fixing $t_{z}$ to an arbitrary constant and adjusting the focal length accordingly, a two-stage approach is proposed, as given in Figure 5.

In the first stage, the “Arbitrary Estimator Network” is used to predict the parameters by eliminating ambiguity. Hence, $t_{z}$ is fixed to an arbitrary constant, and we rescale the focal length accordingly as explained in the previous section. Then, the 5DoF pose and focal length are predicted with $t_{z}$ fixed. Hence, the outputs from Stage I are relative to the fixed $t_{z}$.

Then in the second stage, real $t_{z}$ is predicted and *f* is only scaled up or scaled down proportionally to the update of the $t_{z}$ prediction by the depth estimator network. Unlike the first stage, where an iterative refinement step is employed, using a refiner network similar to [13], the second stage does not employ an iterative refinement step. The main reason is that multiple iterations have been observed to cause overshooting of the predictions. This issue of overshooting will be discussed in the ablation studies section.

#### 3.2.1. Stage I—Arbitrary Estimator Network

The purpose of the “Arbitrary Estimator Network” is to estimate all the parameters with respect to the fixed $t_{z}$ value. The inputs to Stage I are the uncontrolled RGB image I, initial estimates of the 6DoF pose with the focal length, and the corresponding CAD model M. Here, $\theta^{k}$ collectively represents the 3DoF rotation (*R*), the 3DoF translation ($t_{x},t_{y},t_{z}$) along the X,Y,Z axes, and the focal length *f*, as in (17).

(17) $$\theta^{k}=\left\{f,t_{x},t_{y},t_{y},R\right\}$$

As $t_{z}$ is fixed to an arbitrary constant $z_{arb}$, the ground truth of the focal length is rescaled using the relationship explained by Equation (16) during the training phase. The value for $z_{arb}$ can be selected to a near approximate value based on intuition. In the experiments of this study, after analyzing the values of the Pix3D dataset, we set it to 2 m, which is around the median value. The rescaled ground truth values for focal length ${\hat{f}}_{new}$ and pose parameters (${\hat{t}}_{x}$, ${\hat{t}}_{y}$, *R*) with arbitrarily fixed $t_{z}$ to $z_{arb}$ during Stage I are given by Equation (18):

(18) $$\hat{\theta }=\left\{{\hat{f}}_{new},{\hat{t}}_{x},{\hat{t}}_{y},z_{arb},\hat{R}\right\}$$

In the first stage, both coarse and refined steps are devised, where the coarse network estimates an approximate value and the refiner converges toward the target value through multiple iterations. At this stage, the same network described in [13] is used. As a result, the convergence property is inherited from this network, as demonstrated in [5,13]. The CAD model M is rendered using renderer R. $\theta^{k}$ represents the focal length and 5DoF pose components with fixed $t_{z}$ at iteration *k*. A ResNet-50 [41] CNN (convolutional neural network) is employed in both stages. ResNet-50 was selected due to its proven performance [41] in image recognition and feature extraction, providing a good balance between accuracy and computational efficiency. It has also been effectively used in FocalPose, demonstrating its suitability for 6DoF pose estimation tasks. The rendered image and the observed input image I are fed to the CNN F. Here, $\Delta \theta_{k}$ denotes the predicted updates of each pose parameter ($v_{x},v_{y},v_{z},v_{R,1},v_{R,2},v_{R,3}$), and focal length ($v_{f}$) individually, from the network F. The network outputs the values required to update $f,t_{x},t_{y}$ and *R*. The update rule *U* for each parameter θ is given by Equation (19).

(19) $$\theta^{k+1}=U\left(\theta^{k},\Delta \theta_{k}\right)$$

Function *U* is defined individually for each parameter as given below;

- **The 3D translation update rule:** With $\hat{t_{z}}$ fixed to $z_{arb}$, the update rules for the 3D translation in the *x* and *y* directions are given by Equations (20) and (21). $\left[v_{x}^{k},v_{y}^{k}\right]$ represent the outputs predicted for the update of the object’s translation in the x and y directions by the network F, respectively.
  (20) $$t_{x}^{k+1}=\left(\frac{v_{x}^{k}}{f^{k+1}}+\frac{t_{x}^{k}}{z_{arb}}\right)z_{arb}$$
  (21) $$t_{y}^{k+1}=\left(\frac{v_{y}^{k}}{f^{k+1}}+\frac{t_{y}^{k}}{z_{arb}}\right)z_{arb}$$
- **The 3D rotation update rule:** A similar approach to the [13] is used for updating rotations using Gram–Schmidt orthogonalization. The update is performed using the Equation (22):
  (22) $$R^{k+1}=R\left(v_{R,1}^{k},v_{R,2}^{k}\right)R^{k}$$
  where $R_{k+1}$ represents the updated rotation of the object, $R_{k}$ denotes the current rotation, and $R(v_{R,1},v_{R,2})$ is the rotation matrix derived through Gram–Schmidt orthogonalization of the two three-dimensional vectors $v_{R,1}$ and $v_{R,2}$, which are predicted by the alignment network *F* as a component of $\Delta \theta_{k}$.
- **Focal length update rule:** During the training of the first stage, the focal length is rescaled to compensate for setting $t_{z}$ to an arbitrary constant. However, the focal length update rule remains the same as in [13] because there are no correlated parameters in the focal length update rule.
  (23) $$f^{k+1}=e^{v_{f}^{k}}f^{k}$$

The loss function adapted to train Stage I is given by Equation (24). Despite fixing one component ($t_{z}$) of the translation, this stage still estimates the other two components ($t_{x},t_{y}$) of the focal length. This requires the use of two components in the loss function, which include pose loss ($L_{\mathrm{pose}}$) and focal length loss ($L_{\mathrm{focal}}$) for joint learning of pose parameters and focal length during training.

(24) $$L_{stage1}\left(\theta ,{\hat{\theta }}^{\prime }\right)=\alpha L_{\mathrm{focal}}\left(\left(R,t_{x},t_{y},f\right),\left(\hat{R},{\hat{t}}_{x},{\hat{t}}_{y},{\hat{f}}_{new}\right)\right)+L_{\mathrm{pose}}\left(\left(R,t_{x},t_{y}\right),\left(\hat{R},{\hat{t}}_{x},{\hat{t}}_{y}\right)\right)$$

The $L_{\mathrm{focal}}$ component of Equation (24) is described by Equation (25). α and β of Equations (24) and (25) are hyperparameters for the training network. They are calculated using the Huber regression loss and re-projection loss, which are disentangled into the focal length and translation components.

(25) $$\begin{array}{cc} L_{focal} & =\beta L_{H}\left(f,{\hat{f}}_{new}\right)+\\ \; & \frac{1}{2}L_{proj.}\left(\left(R,t_{x},t_{y},{\hat{f}}_{new}\right),\left(\hat{R},{\hat{t}}_{x},{\hat{t}}_{y},{\hat{f}}_{new}\right)\right)+\\ \; & \frac{1}{2}L_{proj.}\left(\left(\hat{R},{\hat{t}}_{x},{\hat{t}}_{y},f\right),\left(\hat{R},{\hat{t}}_{x},{\hat{t}}_{y},{\hat{f}}_{new}\right)\right) \end{array}$$

The Huber regression loss $L_{H}$ measures the errors between the estimated and scaled ground truth focal length using a logarithmic parameterization of focal length as given by Equation (26)

(26) $$L_{H}\left(f,{\hat{f}}_{new}\right)={||log\left(f\right)-log\left({\hat{f}}_{new}\right)\left|\right|}_{H}$$

The other two terms ($L_{proj.}$) in Equation (25) are based on the re-projection error. Instead of directly adding the re-projection error, it is disentangled to separate the effect of error due to focal length estimation and pose parameters. This disentanglement is given by Equation (27). Ref. [13] disentangles this re-projection loss to decouple the effects of the two correlated parameters. However, the update rule involves these correlated parameters.

The projection error $L_{proj}$ measures the difference between the ground truth and the predicted value for each point *p* in the point cloud of the CAD model M. The L1 norm is used to calculate the difference between the projected points of the predicted values and the ground truth. K(f) represents the intrinsic matrix used to project the camera coordinates to the image space.

(27) $$L_{proj.}\left(\left(R,t_{x},t_{y},f\right),\left(\hat{R},{\hat{t}}_{x},{\hat{t}}_{y},{\hat{f}}^{\prime }\right)\right)=\sum_{p\in M}||\pi \left(K\left(f\right),R,t_{x},t_{y},p\right)-\pi \left(K\left({\hat{f}}^{\prime }\right),\hat{R},{\hat{t}}_{x}{\hat{t}}_{y},p\right){\left|\right|}_{1}$$

$L_{\mathrm{pose}}$ of Equation (24) in the loss function of Stage I, represents the disentangled pose loss of the transformed points from the world coordinate system to the camera coordinate system. The mathematical formulation is given by Equation (28). Here, the disentanglement between 2D translation and rotation is considered. As $t_{z}$ is fixed, the effect due to the translation in the *z*-axis in Stage I is not considered.

(28) $$\begin{array}{cc} L_{pose}= & D(U\left(\theta^{k},\left\{v_{x}^{k},v_{y}^{k},z_{arb},{\hat{R}}^{k},{\hat{v}}_{f}^{k}\right\}\right),\hat{R},\hat{t})\\ \; & +D(U\left(\theta^{k},\left\{{\hat{v}}_{x}^{k},{\hat{v}}_{y}^{k},z_{arb},R^{k},{\hat{v}}_{f}^{k}\right\}\right),\hat{R},\hat{t}) \end{array}$$

The distance function D() in Equation (28) is defined using the L1 norm as follows:

(29) $$D\left(\left\{R_{1},t_{1}\right\},\left\{R_{2},t_{2}\right\}\right)=\frac{1}{\left|M\right|}\sum_{p\in M}\left|\right|\left(R_{1}p+t_{1}\right)-\left(R_{2}p+t_{2}\right){\left|\right|}_{1}$$

In Equation (28), $\theta^{k}$ denotes the current estimates of the 6D pose and focal length at iteration *k*. The function *D*, as described in Equation (29), computes the difference between the predicted and ground truth values. The function *U* updates the pose and focal length based on the predicted changes, as in Equation (19).

#### 3.2.2. Stage II: Depth Estimator Network

Stage I generates the camera’s extrinsic and intrinsic parameters relative to the arbitrarily set ($t_{z}$). Hence, the goal of Stage II “Depth Estimator Network” is to estimate the actual depth. Unlike in Stage I, in this stage all the pose parameters are predicted, excluding the focal length. Instead of predicting the focal length, it is scaled based on the predicted value of $t_{z}$.

Similar to the previous stage, a ResNet-50 network is utilized to predict the 6DoF pose. In contrast to Stage I, Stage II does not iteratively refine parameters; instead, it achieves better results with a single forward pass, based on experimental observations given the ablation studies in Section 4.3. The iterative approach in Stage II was found to potentially lead to divergence, likely due to the already refined estimates from Stage I, where iterative refinements were applied.

The prediction of $t_{z}$ (represented by ${t_{z}}^{stage2}$) in this depth estimator network can be represented by Equation (30), where ${t_{z}}^{stage1}$ is the translation along the z-axis predicted by Stage I.

(30) $${t_{z}}^{stage2}=v_{z}{t_{z}}^{stage1}$$

As mentioned previously, instead of predicting the focal length, it is scaled in proportion to the updated $t_{z}$ using the relationship represented by Equation (31). The terms $f^{stage2}$ and $f^{stage1}$ represent the scaled focal length from Stage II and the output focal length predicted by Stage I, respectively.

(31) $$f^{stage2}=f^{stage1}\left(\frac{{t_{z}}^{stage2}}{z_{arb}}\right)$$

Next, the *x* and *y* components of the translation are updated using the following equations:

(32) $${t_{x}}^{stage2}=\left(\frac{v_{x}}{f^{stage2}}+\frac{{t_{x}}^{stage1}}{{t_{z}}^{stage1}}\right){t_{z}}^{stage2}$$

(33) $${t_{y}}^{stage2}=\left(\frac{v_{y}}{f^{stage2}}+\frac{{t_{y}}^{stage1}}{{t_{z}}^{stage1}}\right){t_{z}}^{stage2}$$

This approach ensures a clear transition from the initial estimates to a refined prediction of the camera’s pose and focal length.

In this stage, to train the network, only the pose loss, $L_{\mathrm{pose}}$, is used. $L_{\mathrm{focal}}$ is not used to train the network in Stage II. This is because the focal length is not learned in this stage; instead, it is scaled up or down proportionally to the translation update. Hence, the Stage II loss function can be represented by Equation (34). As 6DoF is computed here, *R* represents 3D rotation and *t* represents 3D translation, which includes $t_{x},t_{y}$, and $t_{z}$.

(34) $$L1_{stage2}=L_{\mathrm{pose}}\left(\left(R,t\right),\left(\hat{R},\hat{t}\right)\right)$$

In contrast to Equation (28), which considered 2D translation, pose loss across 3D translation and 3D rotation is now considered using disentanglement. Hence, the relationship can be expressed by Equation (35)

(35) $$\begin{array}{cc} L_{pose}= & D(U\left(\theta^{old},\left\{v_{x},v_{y},\hat{v_{z}},{\hat{R}}^{k}\right\}\right),\hat{R},\hat{t})\\ \; & +D(U\left(\theta^{old},\left\{{\hat{v}}_{x}^{k},{\hat{v}}_{y}^{k},v_{z},{\hat{R}}^{k}\right\}\right),\hat{R},\hat{t})\\ \; & +D(U\left(\theta^{old},\left\{{\hat{v}}_{x}^{k},{\hat{v}}_{y}^{k},\hat{v_{z}},R^{k}\right\}\right),\hat{R},\hat{t}) \end{array}$$

Here, in Equation (35), *D* is similar to the function explained by Equation (29), which calculates the L1 norm of the distances between transformed ground truth and predicted points. The terms $\left\{v_{x},v_{y},\hat{v_{z}},{\hat{R}}^{k}\right\}$, $\left\{{\hat{v}}_{x}^{k},{\hat{v}}_{y}^{k},v_{z},{\hat{R}}^{k}\right\}$, and $\left\{{\hat{v}}_{x}^{k},{\hat{v}}_{y}^{k},\hat{v_{z}},R^{k}\right\}$ in Equation (35) represents the disentanglement of the pose update across the 2D x−y plane, *z*-axis and rotations, respectively.

To further improve the results, experiments were conducted by introducing the Huber loss of the $t_{z}$ distance in the loss function during the training of Stage II as shown in Equation (36).

(36) $$L2_{stage2}=L_{\mathrm{pose}}\left(\left(R,t\right),\left(\hat{R},\hat{t}\right)\right)+{||log\left(t_{z}\right)-log\left({\hat{t}}_{z}\right)\left|\right|}_{H}$$

As the Huber loss is less sensitive to outliers in data, it is particularly effective in handling transformation errors of the primary objective of predicting $t_{z}$ in the second stage. The introduction of the Huber loss resulted in obtaining better results across several metrics. Importantly, these results are still better than [13], showing the effectiveness of the proposed extended methodology. The experiments indicate that incorporating Huber loss in Stage II yields noticeable improvements over certain metrics. For example, the Huber loss effectively reduces pose and translation errors. However, for some metrics, such as translation accuracy in the Pix3D Chair dataset, the results are comparable with and without Huber loss, yet both still outperform [13]. We believe that this might be due to using real datasets for training [42], without incorporating synthetic data, which introduce greater variability and potential errors.

## 4. Results

### 4.1. Quantitative Results

To evaluate the effectiveness, the proposed method is compared with [13,14] using the Pix3D dataset [42]. Specifically, the Pix3D [42] real dataset was used for the evaluation, without employing the synthetic dataset due to hardware constraints in training. The dataset splits used were the same as in Focalpose [13]: sofa (523 training, 28 validation, 540 test), bed (193 training, 10 validation, 190 test), table (367 training, 19 validation, 351 test), and chair (1431 training, 75 validation, 1387 test). To maintain data sufficiency and enhance generalization, augmentation techniques have been applied during training to increase data size and variability. These techniques included RGB adjustments (blur, sharpness, contrast, brightness, and color), background changes using Pascal VOC dataset [43] backgrounds, and resizing to a 640 × 480 aspect ratio. These augmentations were applied dynamically during data loading at the training stage, ensuring diverse training samples. The training was conducted on an NVIDIA RTX 3090 GPU with each dataset undergoing a thorough training process of 500 epochs, except for the chair dataset, which underwent 200 epochs. It was observed that the chair dataset’s performance stabilized after 150 epochs, hence training was stopped at that stage to optimize resource utilization while ensuring the quality of the results. The value for $t_{z}$ is intuitively selected based on the data distribution of $t_{z}$ values in the Pix3D images of the furniture classes. The effect of arbitrarily setting $t_{z}$ is discussed in the ablation studies.

To assess the performance of the proposed approach, a standard set of evaluation metrics used by [13,14] we used. The following metrics are included in Table 1:

**Median rotation error (*****MedErr.*****)**: Computes the geometric distance between the predicted rotation *R* and the ground truth rotation $\hat{R}$ as given by Equation (37). In the equation, ||F|| represents the Frobenius norm [44], which is defined as the square root of the sum of the absolute squares of its elements.

(37) $$e_{R}=\frac{||\log\left({\hat{R}}^{⊤}R\right){\left|\right|}_{F}}{\sqrt{2}},$$

**Median translation error (*****MedErr.*****)**: Calculates the normalized translation error given by Equation (38), where *t* is the predicted translation and $\hat{t}$ is the ground truth translation. Lower median values indicate more accurate translation predictions.

(38) $$e_{t}=\frac{||t-\hat{t}{\left|\right|}_{2}}{\left|\right|\hat{t}{\left|\right|}_{2}},$$

**Median pose error (*****MedErr.*****)**: This metric measures the median error in the overall pose estimation, combining rotation and translation errors using the point-matching error $e_{R,t}$ in the camera coordinate system as given by Equation (39):

(39) $$e_{R,t}=\frac{d_{\mathrm{bbox}}}{d_{\mathrm{img}}}\underset{p\in M^{⋆}}{\mathrm{avg}}\frac{||\left(Rp+t\right)-\left(\hat{R}p+\hat{t}\right){\left|\right|}_{2}}{\left|\right|\hat{t}{\left|\right|}_{2}},$$

In Equation (39), $d_{\mathrm{bbox}}$ is the diagonal of the ground truth 2D bounding box, $d_{\mathrm{img}}$ is the diagonal of the image, $M^{⋆}$ is the 3D model of the ground truth object instance, *p* represents points in CAD model $M^{⋆}$, (R,t) is the predicted 6D pose, and $\left(\hat{R},\hat{t}\right)$ is the ground truth 6D pose.

**Median focal length error (*****MedErr.*****)**: This metric measures the median error in the focal length estimation. It is calculated as the relative focal length error $e_{f}$ given by Equation (40), where *f* is the predicted focal length and $\hat{f}$ is the ground truth focal length. Lower median values indicate more accurate focal length predictions:

(40) $$e_{f}=\frac{\left|f-\hat{f}\right|}{\hat{f}},$$

**Median projection error (*****MedErr.*****)**: This metric measures the median error in the reprojection of the 3D points into the image plane, taking into account the focal length *f*. It is computed in Equation (41):

(41) $$e_{P}=\underset{p\in M^{⋆}}{\mathrm{avg}}\frac{||\pi \left(R,t,f,p\right)-\pi \left(\hat{R},\hat{t},\hat{f},p\right){\left|\right|}_{2}}{d_{\mathrm{bbox}}},$$

where *p* is a 3D point of the object model $M^{⋆}$, and π(K(f),R,t,p) is the reprojection of *p* using the estimated parameters. Lower median values indicate more accurate reprojections.

**Projection accuracy** (${\mathrm{Acc}}_{P_{0.1}}$, ${\mathrm{Acc}}_{P_{0.05}}$): These metrics represent the percentages of images where the reprojection errors $e_{P}$ are below 0.1 and 0.05 of the image sizes, respectively. Higher percentages indicate better performance.

**Rotation Accuracy at 30°, 15°, 5° (*****Acc 30°***, ***Acc 15°***, ***Acc 5°*****)**: These metrics represent the percentage of images for which the rotation error $e_{R}$ is within 30°, 15°, and 5°, respectively. Higher percentages indicate better performance.

Table 1 demonstrates the results of experiments conducted in both Stage I and Stage II (with the two loss functions of $L1_{stage2}$ and $L2_{stage2}$), compared with [13,14]. The results demonstrate significant improvements across multiple evaluation metrics.

While Focalpose++ [14] demonstrates improved performance over Focalpose [13] across most evaluated metrics, the methodology proposed in this work achieves outstanding results in nearly all metrics compared with both works. However, there are certain metrics, such as in the Pix3D Sofa category where it does not perform as well. On average, Stage I of this study increases projection accuracy by 31.86% and decreases the median error of focal length by 75.89% compared to Focalpose++. This significant reduction in focal length error is due to fixing the $t_{z}$ value to an arbitrary constant and estimating only six parameters in Stage I. By setting the z-axis translation as a constant, the proposed method simplifies the problem and achieves more precise translation predictions, leading to a noticeable decrease in median translation error across all datasets. Stage I performs exceptionally well with this reduced parameter set, making it suitable for applications where depth detail is intuitive and can be estimated.

Stage II of the proposed approach decreases the median projection error by 9.37% and 7.19% with the L1 and L2 loss functions, respectively. Additionally, there is a significant decrease in the median translation error, which is 17.56% and 20.27% using L1 and L2, respectively. Stage II is suitable for applications requiring full parameter estimation. As the inputs to Stage II are the outputs from Stage I, the performance of Stage II relies on Stage I. However, there is still a chance for improvement in Stage II. For example, projection accuracy estimations for the Sofa class in Stage II using $L1_{stage2}$ are lower compared to [14]. We believe the proposed method will perform better with training using noise-free synthetic data.

Experiments demonstrate that the inclusion of the Huber loss in the Stage II loss function (36) results in improvements across several metrics. On average, the average median translation error and median focal length error across all the Pix3D classes have been reduced by 2.79% and 0.41% respectively. This is factored by the addition of a translation component to the loss function, improving overall transformation accuracy. These reductions highlight the robustness of the Huber loss in handling outliers and improving prediction accuracy. However, there were no significant reductions in the median translation and pose error in the Pix3D Chair class and also across several metrics as shown by Table 1, though still better than the benchmark set by FocalPose. This inconsistency might be attributed to only using real datasets for training, which adds more variability and potential errors compared to synthetic data.

### 4.2. Qualitative Results

**Figure 6:**
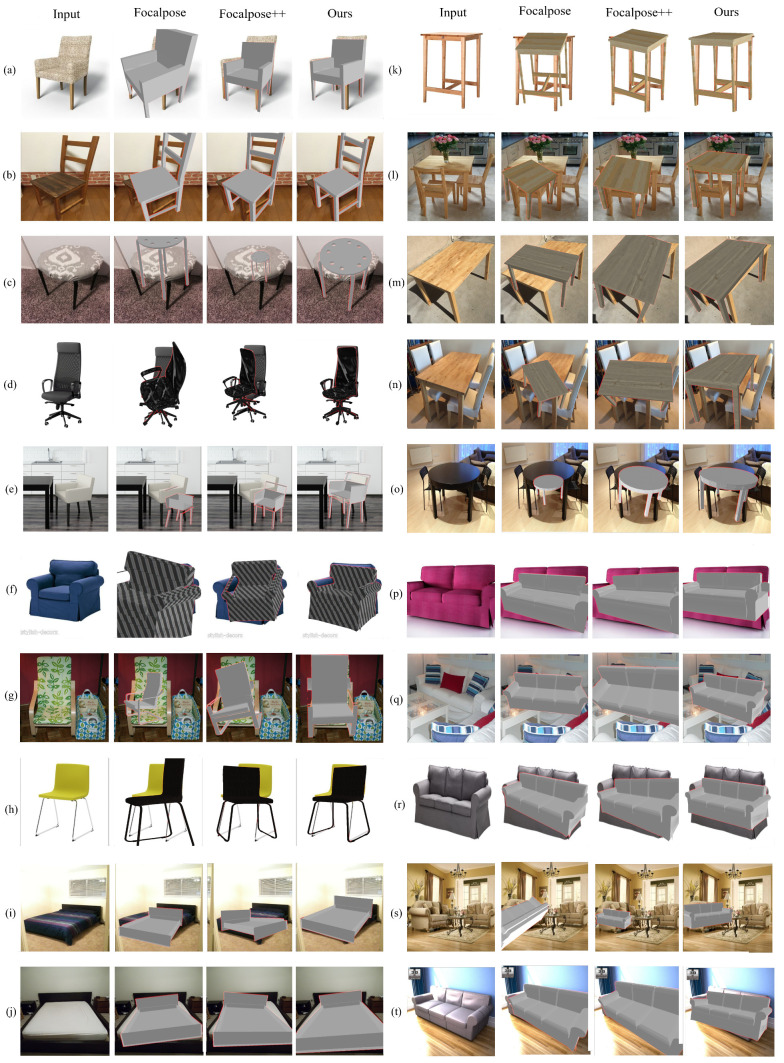
Comparison of the outputs from the proposed method with Focalpose [13] and Focalpose++ [14] using Pix3D dataset. Subfigures (**a**–**t**) represents different classes of chair, sofa, bed and table of Pix3D Dataset. Metadata of these images are not available during the inference time.

Figure 6 presents a qualitative comparison of the results of the proposed method with Focalpose and Focalpose++. These images are obtained from the Pix3D dataset [42], and the focal length details are not available during the inference time When observing the results given in the figure, it can be seen that the proposed approach achieves more accurate scaling of the CAD model rendering on real-world RGB images compared to [13,14]. This improvement is primarily due to the proposed strategy of decoupling the correlated parameters, which simplifies the complexity of the estimation. When the z-axis translation and focal length are updated simultaneously during prediction, it often results in locally optimal solutions, as shown in Figure 6b–d,f,g,o,s, leading to scaling issues. In contrast, the proposed approach yields better renderings in terms of projection accuracy.

Consequently, the qualitative results presented in this work validate the effectiveness of the proposed method in addressing the challenges associated with pose estimation and focal length prediction, demonstrating significant improvements over [13], especially in terms of projection accuracy and model scaling. The ability to perform well without prior metadata underlines the robustness and practicality of the approach in real-world scenarios where such metadata are often unavailable.

### 4.3. Ablation Study

#### 4.3.1. Effect of Using a Refiner in Stage II

**Figure 7:**
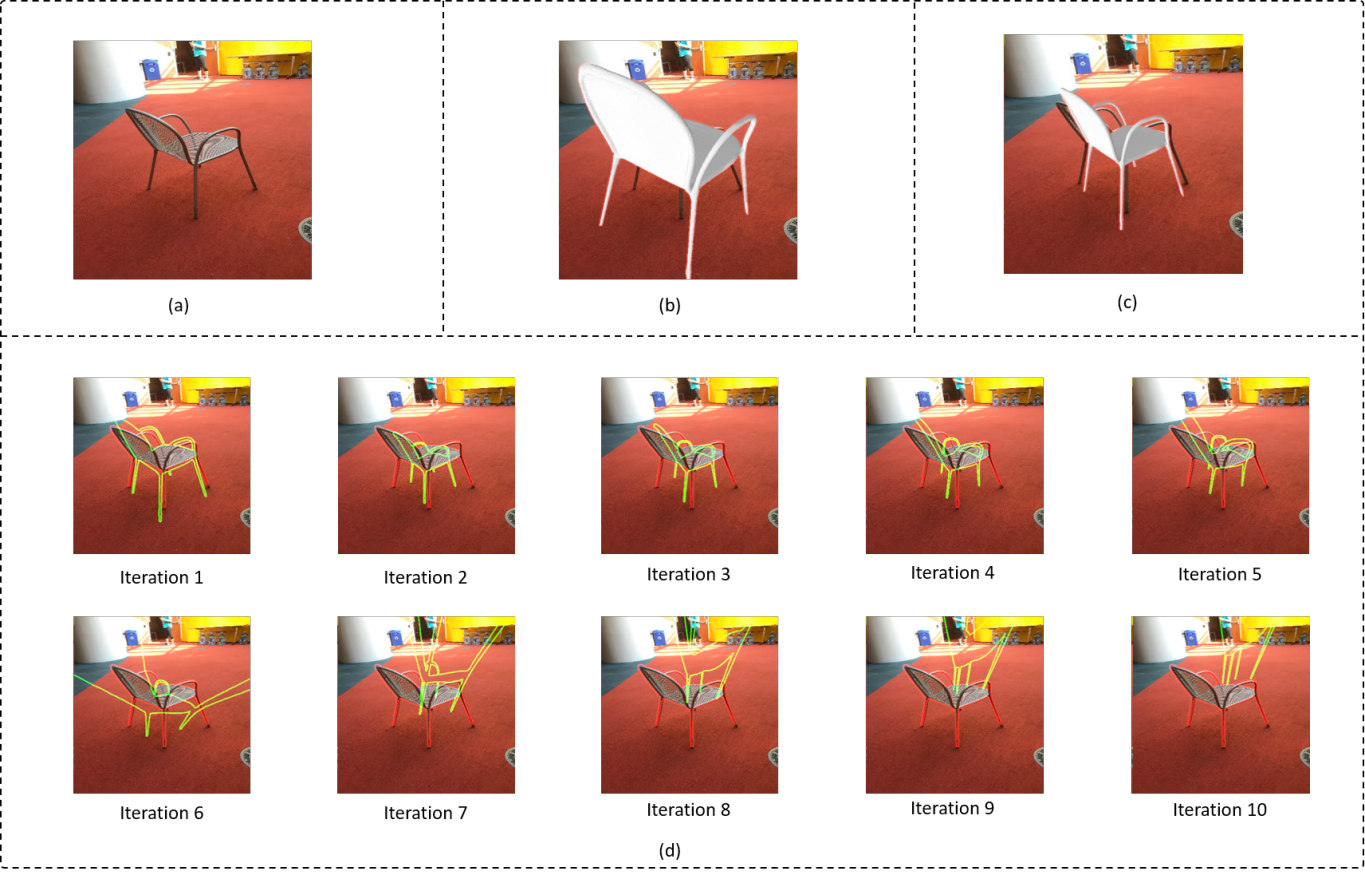
(**a**) Input single RGB image, (**b**) prediction from Focalpose [13], (**c**) prediction from the proposed work (Stage II output), (**d**) outputs by employing multiple refiner iterations to Stage II of the proposed approach. The green-colored contours represent the predicted pose during each iteration in the refiner of Stage II, and the red colored contour represent the ground truth.

In this section, similar to Stage I, an experiment was conducted incorporating an iterative refinement network in Stage II. The results of these experiments indicate that there is a divergence in the network output. This divergence may be caused by the already existing refinement process in Stage I, which causes behavior similar to an exploding gradient when values are already near convergence. Although a prediction relative to a fixed $t_{z}$ is done in Stage I, $t_{x}$, $t_{y}$, and the rotational components are converged close to the target values due to the refiner in Stage I. Consequently, Stage II is primarily focused on estimating $t_{z}$ while adjusting the focal length. The usage of another refiner at this stage is likely to cause the previously converged values to diverge. In Figure 7, the divergence effect caused by multiple refiner iterations in the Stage II network is clearly seen. As the number of iterations increases, the predicted position and orientation (shown by the green-colored contour) are diverged from the accurate prediction of Stage I.

#### 4.3.2. Effect of Loss Functions

Experiments on the other possible loss functions for Stage II were also conducted to evaluate their impact on the metrics. The effect of including versus excluding the projection error in the loss function of Stage II was experimented with. This study was conducted using the Pix3D Bed dataset. As shown in Table 2, the performance is better when the projection error is not included in the loss function in Stage II. This improvement is based on the reason that Stage II focuses solely on the camera coordinate space and not the image coordinate space (as the focal length is not predicted but scaled in Stage II). Hence, only the transformation error ($L_{\mathrm{pose}}$) directly relates to the spatial arrangement of the camera and the object in the camera space. By excluding the projection error, the model can optimize the camera parameters without being influenced by discrepancies in the image space.

#### 4.3.3. Effect of Selection of $t_{z}$ Value in Stage I

During Stage I, the value of $t_{z}$ is initially fixed to an arbitrary constant to simplify the 6DoF pose estimation problem. The choice of this value is important as it can influence the accuracy of the pose and focal length predictions. Through a set of experiments, the effect of different $t_{z}$ values on the overall performance was analyzed.

**Figure 8:**
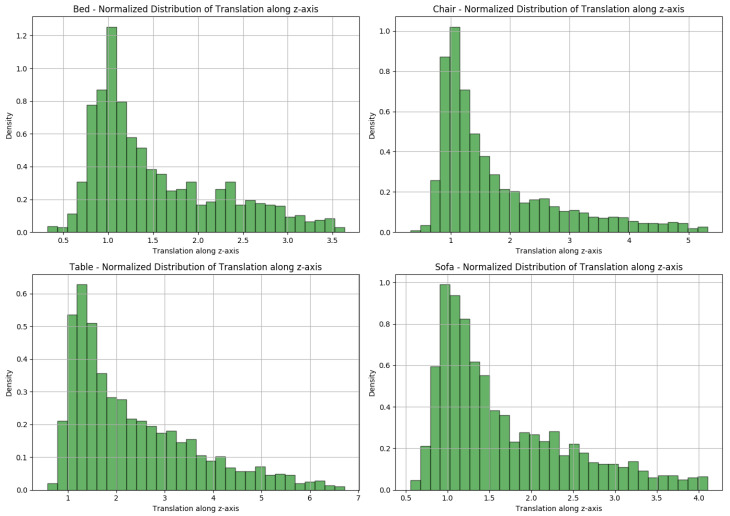
Distribution of $t_{z}$ across different classes in Pix3D dataset.

In Figure 8, the normalized distribution of the filtered $t_{z}$ values for each category of Pix3D was plotted to visualize the spread and central tendency of the data. These distributions are very useful for understanding the typical range of translations in the Pix3D Dataset.

The $t_{z}$ value was chosen based on the observed median values of the filtered translation along the z-axis in the Pix3D dataset. A filtering process was applied by removing outliers using the interquartile range (IQR) method. After this filtering, the mean and median $t_{z}$ values for four classes (bed, chair, table, and sofa) were calculated. The results are summarized in Table 3.

Based on these observations and the distribution of values, $t_{z}=2$ m was selected as a reasonable and approximate value that lies as a rounded value for the range of these categories. Based on the experimental results shown in Table 4, it is proven that this value serves as a good initialization point for the translation along the z-axis.

To assess the impact of different $t_{z}$ values, experiments were conducted with values that were too small (0.2 m), near the mean and median (2 m), and excessively large (20 m) on the Pix3D bed class. The results are summarized in Table 4.

From the results, it is proved that:

- Small $t_{z}$ (0.2 m): This value resulted in relatively high translation and focal length estimation errors.
- Optimal $t_{z}$ (2 m): This value produced the best balance, with lower median errors in translation and focal length, and also a higher projection accuracy. This validates the choice of 2 m as a good approximation for initialization.
- Large $t_{z}$ (20 m): This value degraded the performance, with comparatively higher errors in focal length estimation and lower projection accuracy.

## 5. Discussion

While this study presents promising results across most of the evaluation metrics, it has several limitations. The dependency only on real datasets for training introduces variability and potential errors that may affect performance due to the noisiness in data. The proposed method can be improved in projection accuracy for specific classes such as Pix3D Sofa. Sensitivity to real-world noise suggests that incorporating noise-free synthetic data could enhance robustness. The training was performed on real data due to hardware constraints. The training time for the largest dataset, the Pix3D Chair class, nearly takes 30 h for Stage I and 17 h for Stage II. After training Stage I, the outputs should be used as inputs for Stage II training, which requires running inference on all the datasets for another 30 h. Therefore, considering the total time for the Pix3D Chair dataset alone, it takes around 70–80 h to complete an end-to-end experiment on a single NVIDIA RTX GPU.

The choice of the $t_{z}$ value is critical, as it influences directly pose and focal length accuracy. While this study presents a rationale for the selected 2-m initialization value based on intuition and data distribution, different $t_{z}$ values can significantly impact performance. Experiments showed that small or excessively large $t_{z}$ values degrade performance, highlighting the need for an optimal $t_{z}$ setting which is a near-approximate. The proposed method relies on the assumption that an approximate depth can be intuitively estimated based on contextual information or user input. This dependency could limit the method’s effectiveness in scenarios where such intuitive estimation is not feasible

It is important to note that, when compared to state-of-the-art pose estimators such as [4,5], which consider the focal length as a prerequisite, the results of Stage II of the proposed approach along with Focalpose and Focalpose++ do not achieve the same level of accuracy in renderings. This discrepancy is expected, given that the problem domain involves the additional complexity of estimating the focal length alongside the 6DoF pose, making the problem inherently more complex.

## 6. Conclusions

This study presents a novel two-stage method for estimating 6DoF object poses and focal lengths from single RGB images obtained in uncontrolled environments. The approach addresses the projection scale ambiguity that arises from the correlation between the z-axis translation ($t_{z}$) and the camera’s focal length (*f*), and decouples these parameters to enhance the accuracy of pose and focal length estimation.

In the first stage, the z-axis translation is fixed to an arbitrary value, simplifying the estimation process for the other pose parameters and the focal length. This initial simplification provides a foundation for more accurate predictions. In the second stage, the true value of the z-axis translation is predicted, and the focal length is adjusted accordingly. This two-stage approach significantly reduces projection errors, as demonstrated by experimental results on benchmark datasets.

Validation using the Pix3D real dataset shows substantial improvements compared to state-of-the-art methods of Focalpose and Focalpose++. On average, Stage I of this study increases projection accuracy by 31.86% and decreases the median error of focal length by 75.89% compared to FocalPose++. Stage II of the proposed approach decreases the median projection error by 9.37% and 7.19% with the L1 and L2 loss functions, respectively. Additionally, there is a significant decrease in the median translation error, which is 6.16% and 6.65% using L1 and L2, respectively. When comparing the two loss functions of Stage II L1 and Stage II L2, the introduction of Huber loss to Stage II loss function decreases the average median translation error by 2.79% and the average median focal length error by 0.41% across the Pix3D classes.

This method demonstrates significant improvements in 6DoF pose estimation using uncontrolled RGB images, providing a reliable solution for applications in extended reality (XR), robotics, and 3D object tracking. Future research may focus on refining the model by incorporating synthetic datasets and exploring additional loss functions to improve performance across various scenarios. Additionally, incorporating a separate depth estimation pipeline to initialize a value for $t_{z}$ could further enhance the applicability of the proposed method.

## Figures and Tables

**Table 1:** Comparison of the proposed approach (Stage I, Stage II with L1, and Stage II with L2) with FocalPose [13] and Focalpose++ [14].

Dataset	Method	DoF	Rotation					Translation		Pose		Focal Length		Projection		
			MedErr. ↓	*Acc 30°* ↑	*Acc 15°* ↑	*Acc 5°* ↑		MedErr. ↓		MedErr. ↓		MedErr. ↓		MedErr. ↓	${\mathrm{Acc}}_{P_{0.1}}$ ↑	${\mathrm{Acc}}_{P_{0.05}}$ ↑
Pix3D Bed	FocalPose [13]	7	0.436	53.68%	32.11%	3.16%		0.251		0.202		0.222		0.132	41.05%	13.16%
	FocalPose++ [14]	7	0.450	53.68%	37.37%	7.37%		0.204		0.176		0.204		0.135	40.53%	**18.95%**
	Proposed (Stage I)	6	**0.389**	**62.11%**	**37.89%**	6.32%		**0.019**		**0.044**		**0.064**		**0.104**	**47.37%**	**20.53%**
	Proposed (Stage II-L1)	7	**0.382**	**60.00%**	36.32%	**7.89%**		0.200		0.179		0.208		**0.119**	**45.26%**	18.42%
	Proposed (Stage II-L2)	7	0.387	57.37%	**39.47%**	6.84%		**0.187**		**0.174**		**0.199**		0.129	44.21%	17.89%
Pix3D Sofa	FocalPose [13]	7	0.236	79.78%	56.77%	10.39%		0.230		0.153		0.208		0.057	74.77%	43.04%
	FocalPose++ [14]	7	0.193	90.74%	69.26%	11.48%		0.203		0.137		0.195		**0.048**	**81.85%**	**53.89%**
	Proposed (Stage I)	6	**0.134**	**94.07%**	**80.37%**	**30.56%**		**0.012**		**0.017**		**0.038**		**0.038**	**87.04%**	**65.37%**
	Proposed (Stage II-L1)	7	**0.169**	**92.02**%	**74.21%**	20.04%		0.200		0.132		**0.194**		0.056	81.45%	41.19%
	Proposed (Stage II-L2)	7	0.172	91.47%	73.10%	**20.59%**		**0.192**		**0.124**		0.197		0.055	81.82%	43.97%
Pix3D Table	FocalPose [13]	7	0.762	36.75%	17.38%	1.71%		0.503		0.312		0.323		0.204	19.09%	3.70%
	FocalPose++ [14]	7	0.617	42.17%	21.08%	2.28%		0.391		0.277		0.363		0.202	23.36%	6.84%
	Proposed (Stage I)	6	**0.500**	**51.28%**	**27.07%**	3.70%		**0.021**		**0.053**		**0.075**		**0.136**	**38.46%**	**15.38%**
	Proposed (Stage II-L1)	7	**0.587**	**47.29%**	**26.50%**	4.56%		0.279		0.213		**0.315**		**0.180**	27.07%	**7.41**%
	Proposed (Stage II-L2)	7	0.611	46.44%	24.50%	**5.13%**		**0.272**		**0.211**		0.320		0.182	26.21%	5.70%
Pix3D Chair	FocalPose [13]	7	0.964	24.08%	7.47%	0.44%		0.553		0.376		0.210		0.182	16.17%	1.45%
	FocalPose++ [14]	7	0.594	45.35%	20.12%	1.66%		0.348		0.229		0.242		0.137	35.11%	9.88%
	Proposed (Stage I)	6	**0.278**	**66.69%**	**47.95%**	**7.86%**		**0.020**		**0.026**		**0.061**		**0.068**	**62.44%**	**35.26%**
	Proposed (Stage II-L1)	7	0.288	**66.35%**	44.96%	7.40%		**0.216**		**0.146**		**0.210**		**0.096**	**51.56%**	20.96%
	Proposed (Stage II-L2)	7	**0.286**	66.28%	**46.41%**	**7.54%**		0.220		0.147		0.211		0.098	50.69%	**21.25%**

7-DoF: 6-DoF pose + focal length. 6-DoF: 2-DoF translation + 3-DoF rotation + focal length. Proposed (Stage II-L1) represents the results from the complete pipeline with $L1_{stage2}$ as a loss function (Equation (34)). Proposed (Stage II-L2) represents the results from the complete pipeline with $L2_{stage2}$ as a loss function (Equation (36)). **Note:** The bold values indicate the best results of the Proposed Stage I when compared with FocalPose and FocalPose++, and the best results of the Proposed Stage II (with L1 and L2) when compared with FocalPose and FocalPose++. The comparisons for Stage I and Stage II are conducted independently. The symbols “↑” and “↓” indicate that higher and lower values are better under each metric, respectively.

**Table 2:** Ablation study results: impact of including the projection error in the loss function of Stage II.

Parameter	Metric	$L_{\mathrm{stage}2}$ = $L_{\mathrm{pose}}$	$L_{\mathrm{stage}2}$ = $L_{\mathrm{pose}}$ + $L_{\mathrm{proj}}$
Rotation	MedErr. ↓	**0.3821**	0.4305
	***Acc 30°*** ↑	**0.6000**	0.5737
	***Acc 15°*** ↑	**0.3632**	0.3421
	***Acc 5°*** ↑	**0.0789**	0.0474
Translation	MedErr. ↓	**0.1997**	0.2451
Focal	MedErr. ↓	**0.2084**	0.2961
Pose	MedErr. ↓	**0.1788**	0.1954
Projection	MedErr. ↓	**0.1189**	0.1239
	${\mathrm{Acc}}_{P_{0.1}}$ ↑	**0.4526**	0.4211
	${\mathrm{Acc}}_{P_{0.05}}$ ↑	**0.1842**	0.1684

**Note:** The bold values indicate the outperforming values under each metric. The symbols “↑” and “↓” indicate that higher and lower values are better under each metric, respectively.

**Table 3:** Mean and median $t_{z}$ values for different categories in the Pix3D dataset.

Category	Mean (m)	Median (m)
Bed	1.53	1.27
Chair	1.77	1.35
Table	2.35	1.93
Sofa	1.67	1.39

**Table 4:** Impacts of different $t_{z}$ values on performance metrics on the Pix3D bed class.

Parameter	Metric	$t_{z}=0.2$ m	$t_{z}=2$ m	$t_{z}=20$ m
Rotation	MedErr. ↓	0.3286	0.3893	**1.1300**
	***Acc 30°*** ↑	0.5947	**0.6211**	0.1053
	***Acc 15°*** ↑	**0.4158**	0.3789	0.0158
	***Acc 5°*** ↑	**0.0632**	**0.0632**	0.0053
Translation	MedErr. ↓	0.1554	**0.0185**	0.0217
Focal	MedErr. ↓	0.1325	**0.0641**	0.0985
Pose	MedErr. ↓	0.3445	**0.0440**	0.0116
Projection	MedErr. ↓	0.2102	**0.1040**	0.2416
	${\mathrm{Acc}}_{P_{0.1}}$ ↑	0.2053	**0.4737**	0.1053
	${\mathrm{Acc}}_{P_{0.05}}$ ↑	0.0368	**0.2053**	0.0158

**Note:** The bold values indicate the outperforming values under each metric. The symbols “↑” and “↓” indicate that higher and lower values are better under each metric, respectively.

## Data Availability

The data presented in this study are available upon request from the corresponding author.
